# Diversity of metalloproteinases in *Bothrops neuwiedi *snake venom transcripts: evidences for recombination between different classes of SVMPs

**DOI:** 10.1186/1471-2156-12-94

**Published:** 2011-11-01

**Authors:** Ana M Moura-da-Silva, Maria Stella Furlan, Maria Cristina Caporrino, Kathleen F Grego, José Antonio Portes-Junior, Patrícia B Clissa, Richard H Valente, Geraldo S Magalhães

**Affiliations:** 1 Laboratório de Imunopatologia, Instituto Butantan, São Paulo, São Paulo, Brazil; 2 Laboratório de Herpetotologia, Instituto Butantan, São Paulo, São Paulo, Brazil; 3 Laboratório de Toxinologia, Instituto Oswaldo Cruz (Fiocruz), Rio de Janeiro, Brazil

## Abstract

**Background:**

Snake venom metalloproteinases (SVMPs) are widely distributed in snake venoms and are versatile toxins, targeting many important elements involved in hemostasis, such as basement membrane proteins, clotting proteins, platelets, endothelial and inflammatory cells. The functional diversity of SVMPs is in part due to the structural organization of different combinations of catalytic, disintegrin, disintegrin-like and cysteine-rich domains, which categorizes SVMPs in 3 classes of precursor molecules (PI, PII and PIII) further divided in 11 subclasses, 6 of them belonging to PII group. This heterogeneity is currently correlated to genetic accelerated evolution and post-translational modifications.

**Results:**

Thirty-one SVMP cDNAs were full length cloned from a single specimen of *Bothrops neuwiedi *snake, sequenced and grouped in eleven distinct sequences and further analyzed by cladistic analysis. Class P-I and class P-III sequences presented the expected tree topology for fibrinolytic and hemorrhagic SVMPs, respectively. In opposition, three distinct segregations were observed for class P-II sequences. P-IIb showed the typical segregation of class P-II SVMPs. However, P-IIa grouped with class P-I cDNAs presenting a 100% identity in the 365 bp at their 5' ends, suggesting post-transcription events for interclass recombination. In addition, catalytic domain of P-IIx sequences segregated with non-hemorrhagic class P-III SVMPs while their disintegrin domain grouped with other class P-II disintegrin domains suggesting independent evolution of catalytic and disintegrin domains. Complementary regions within cDNA sequences were noted and may participate in recombination either at DNA or RNA levels. Proteins predicted by these cDNAs show the main features of the correspondent classes of SVMP, but P-IIb and P-IIx included two additional cysteines cysteines at the C-termini of the disintegrin domains in positions not yet described.

**Conclusions:**

In *B. neuwiedi *venom gland, class P-II SVMPs were represented by three different types of transcripts that may have arisen by interclass recombination with P-I and P-III sequences after the divergence of the different classes of SVMPs. Our observations indicate that exon shuffling or post-transcriptional mechanisms may be driving these recombinations generating new functional possibilities for this complex group of snake toxins.

## Background

In some reptiles and other animals, protein recruiting to specialized glands and their diversification throughout evolution resulted in venoms, which represent a key evolutionary innovation used by these animals for defense, competitor deterrence or predation [1]. When inoculated into a prey/predator, these proteins target important physiological pathways and tissue types. Toxins impairing prey motility have been particularly selected during this process being the neurotoxins available in almost all taxons of venomous animals. On the other hand, the distribution of toxins by the bloodstream makes the hemostatic system also an important venom target. Despite the functional versatility of venom proteins, only a few protein families are frequent in venom composition and this aspect is compensated by a high degree of sequence variability, within the superfamily, generated by accelerated evolution [2].

In viper snake venoms, the most abundant protein families are serine proteinases, metalloproteases, L-amino acid oxidases, phospholipases A_2 _(PLA_2_s), disintegrins, C-type lectins, myotoxins, and CRISP toxins [3], represented by a number of distinct copies with variable structural motifs and functional activities. To explain the generation of such diversity, evolutionary relationships of venom PLA2s [4,5], C-type lectin-like proteins [6], serine proteinase inhibitors [7], and metalloproteinases [8] described that these different sequences evolved by gene duplications followed by positive selection of non-synonymous mutations accumulated in the genomes under strong positive adaptive selection. High levels of variation in venom proteins would confer an advantage to adapt to a variety of different prey [9,10]. Besides the diversity generated during evolution, sequence variability may further increase during post-translational modifications of nascent toxins [11] and by differential expression of toxin groups according to snake geographical distribution [12], sex [13] or ontogenesis [14,15]. For example, in Costa Rica, toxin profiles of *Bothrops asper *venom from adult snakes are characterized by the predominance of PI-SVMPs and K49-PLA2s [16] whereas in Brazil, venom of adult *Bothrops atrox *present the paedomorphic phenotype with predominance of PIII-SVMPs and D49-PLA2s [17]. The paedomorphic phenotype is prevalent in the most important species of *Bothrops *snakes in Brazil, with predominance of P-III SVMPs in venom proteomes or transcriptomes [18-20].

SVMPs are encompassed in the M12 subfamily of metalloproteinases (reprolysins). They are composed of an independent proteolytic domain or of combination between the proteolytic domain and additional adhesive/anti-adhesive domains, as disintegrin or disintegrin-like/cysteine-rich domains, preceded by a N-terminal well conserved pro-domain. Following different steps of post-translational modifications, distinct types of SVMPs may be found in viper venoms, being originally derived from distinct precursor molecules. SVMPs were formerly classified as four distinct classes of nucleotidic sequences (N-classification) that after translation would give rise to four classes of mature proteins (P-classification) with different domain organization. Precursors belonging to class N-I (pre-, pro- and catalytic domains), N-II (pre-, pro-, catalytic and disintegrin domains), N-III (pre-, pro-, catalytic, disintegrin-like and cysteine-rich domains) and N-IV (pre-, pro-, catalytic, disintegrin-like, cysteine-rich and lectin-like domains) would allow biosyntheses of P-I to P-IV SVMPs that include the same domains present on precursors, but with removal of pre- and pro-domains. According to this classification, catalytic domains of P-II, P-III and P-IV SVMPs could also be proteolytically removed to generate P-I SVMPs [21]. However, N-IV precursors have never been found in gland transcriptomes and independent catalytic domains of P-III or P-IV SVMPs have never been isolated as a P-I SVMP in snake venoms. Therefore, classification of SVMPs was reviewed several times and what is currently accepted is the same terminology for N- or P- classes, now referred only as P-classes, and inclusion of the N/P-IV class as a post-translational modification of a class P-III SVMP, presently classified as P-IIId [11]. It is now accepted 11 different subclasses of SVMPs with the following distribution: one type in class P-I, 6 in class P-II and 4 in class P-III, each generating different possibilities of mature proteins depending on dimerization or proteolytic release of independent disintegrin or disintegrin-like domains [11]. The presence and structural features of the domains characterize the function and toxicity of SVMPs. In this regard, SVMPs play important roles in hemostatic disorders and local tissue damage that follows snakebite showing diverse effects both on soluble factors and cellular components. The action of SVMPs involves catalytic and adhesive/anti-adhesive properties, as well as direct cellular activation and/or the release of endogenous bioactive components. They hydrolyze extracellular matrix, plasma and cell-surface proteins, interfere with blood coagulation and fibrinolysis, inhibit platelet aggregation, and show a diversity of activities towards inflammatory and endothelial cells compromising the integrity of capillary vessels [22].

To understand the structural complexity and functional diversity of SVMPs, some studies attempted to explain the recruitment and evolution of this protein family within venom glands of viper snakes. In early studies, we suggested a common ancestry of mammalian matrix metalloproteinases (MMPs) with a P-III-type of SVMP that underwent divergence after gene duplications generating P-II and P-I classes scaffolds [8]. Further studies attempted to elucidate the evolutionary history of SVMPs and one important aspect that has been speculated is that loss of domains in the P-III ancestor gene could be attributed to the neofunctionalization of the disintegrin domain in some of the duplicated P-III genes [23,24]. Recently, Casewell et al. [25] proposed that the evolution of SVMPs is repeatedly punctuated by domain loss to form multiple P-I and P-II SVMP structures. However, it remains untested if the gain of domain structures through mechanisms as exon shuffling or pre- or post-transcriptional recombination could also play a role in the diversification of SVMP structures.

In this study, we have unveiled the diversity of SVMPs expressed by a specimen of *Bothrops neuwiedi *snake by cloning, sequencing and analyzing the SVMP cDNAs transcribed in a single venom gland. A great diversity in the SVMP repertoire was confirmed. Moreover, evidences emerged for recombination between genes of SVMP sequences from different classes and also between their primary mRNA transcripts, enlarging the possibilities of mechanisms involved in generation of diversity of this class of toxins and defining new perspectives in the classification and function prediction of this group of toxins.

## Results

### Diversity of SVMP sequences in Bothrops neuwiedi venom

**Figure 1:**
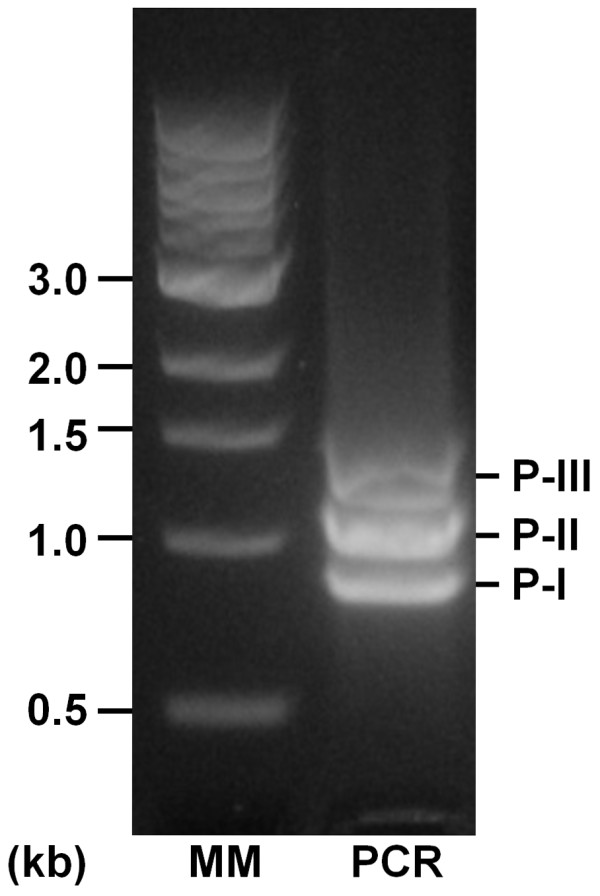
**Amplification of SVMP cDNAs by PCR**. Total cDNA obtained by reverse transcription of venom gland isolated mRNA was submitted to PCR using as primers PRODOM and 3'UTR as described in Methods. Samples were analyzed in 1.5% agarose gels stained with GelRedTM (Biotium). (MM) Molecular markers Biolab ladder; (PCR) amplification product. Note the bands with expected sizes of SVMPs classes P-I, P-II and P-III.

In this study, thirty-one cDNA regions coding for mature SVMPs, isolated from a single venom gland of *Bothrops neuwiedi *snake, were completely sequenced and compared. Total RNA was isolated from the venom gland and SVMP cDNAs were amplified using primers corresponding to conserved regions flanking the segment coding for mature SVMPs in P-I, P-II and P-III classes. At the first cDNA amplification, three distinct bands between 700 and 1500 bp were detected and the more intense was the fraction of medium size that corresponds to P-II SVMPs (Figure 1). These bands were cut from the agarose gels and independently cloned into pGEM-T vector for sequencing. After complete sequencing of the inserts, cDNAs were classified according to size, seven sequences corresponding to PI, ten to P-II and four to P-III SVMPs. Amongst P-II cDNAs, two clones presented interesting unique sequences and therefore were submitted to further sequencing by independent cDNA amplification using other primers to confirm different types of P-II SVMPs in the venom gland generating ten more clones. Out of the thirty-one clones sequenced, identical cDNAs were grouped in eleven distinct sequences of SVMPs which were named according to the snake species (Bn), toxin function (MP), class (I, II or III), subclass (a, b or x) and isoform (1, 2 or 3) as depicted in Table 1. These sequences were deposited in the GenBank under accession numbers HM443632 to HM443642.

**Table 1:** cDNAs characterized from *Bothrops neuwiedi *venom gland.

*Common Name*	*Accession Numbers*	*Lenght (bp)*	*SVMP class*	*Clones sequenced (number)*
BnMP-I_1_	HM443635	777	P-I	2

BnMP-I_2_	HM443636	826	P-I	5

BnMP-IIa	HM443642	1031	P-II	8

BnMP-IIb_1_	HM443637	1053	P-II	4

BnMP-IIb_2_	HM443638	975	P-II	1

BnMP-IIx_1_	HM443639	1068	P-II	3

BnMP-IIx_2_	HM443640	991	P-II	2

BnMP-IIx_3_	HM443641	991	P-II	2

BnMP-III_1_	HM443632	1360	P-III	2

BnMP-III_2_	HM443633	1357	P-III	1

BnMP-III_3_	HM443634	1360	P-III	1

### Sequence alignments predict rearrangements of cDNAs

**Figure 2:**
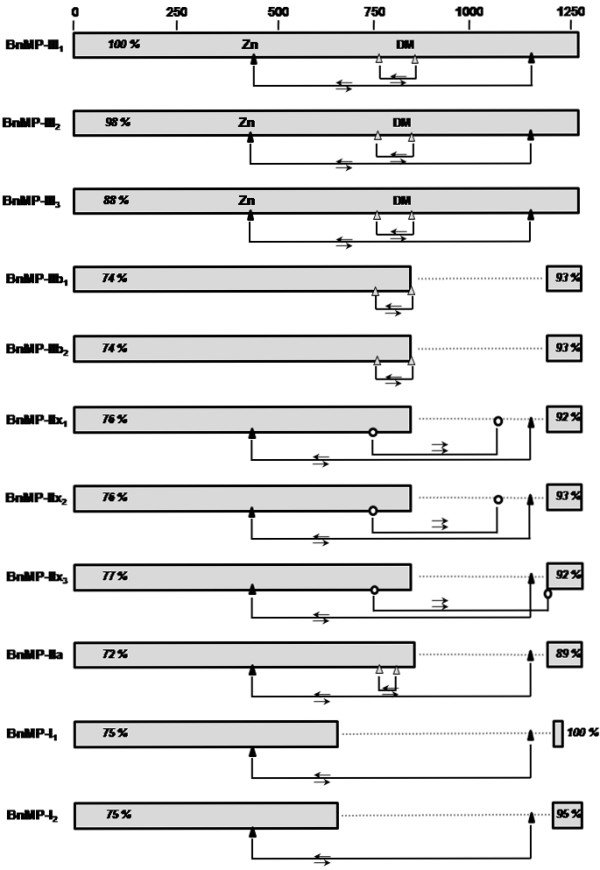
**Schematic representation of sequence alignment of cDNAs encoding *B. neuwiedi *SVMPs**. Complete cDNA sequences were aligned using the Blastn program for multiple sequences alignments using as query BnMP-III_1 _sequence (additional file 1). Boxes represent similarity regions (5'UTR plus mature protein coding regions or 3'UTR) with %identities in relation to the query. Triangles represent positions of complementary regions (⇆) and circles represent the repeated regions (⇉) relatively to BnMP-III_1 _sequence. DM corresponds to the approximate position of the RGD or ECD motifs in disintegrins or disintegrin-like domains, respectively. Dots represent the gaps corresponding to cysteine-rich or disintegrin-like/cysteine-rich domains introduced to maximize sequence alignments.

Sequences were aligned and analyzed according to general domain structure, presence of repeats within the domains and phylogenetic relationships with SVMPs from other snake venoms. Figure 2 shows a schematic representation of the eleven sequences alignment using as query the longest sequence, BnMP-III_1_. The complete alignment is shown in the additional file 1. BnMP-III sequences showed the length of other P-III SVMPs, with approximately 1250 bp coding for the mature protein. A gap corresponding to the C-terminus of disintegrin domain and the entire cysteine-rich domain was evidenced in BnMP-II sequences. In BnMP-I sequences this gap included the entire disintegrin-like and cysteine-rich domains (dotted lines). Figure 2 also shows %identities within the sequences compared to BnMP-III_1_. Similarities confirmed the expected divergence between BnMP-I, BnMP-II and BnMP-III sequences with %identities varying from 88% among SVMPs class P-III and 72 to 77% between P-III and P-I or P-II SVMPs. Interestingly, the identities of 3'UTRs (untranslated regions) among all sequences were more than 90%.

**Figure 3:**
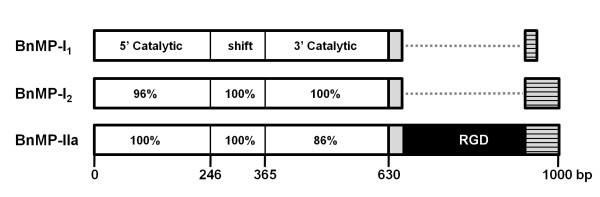
**Schematic representation of sequence alignment of cDNAs encoding BnMP-I_1_, BnMP-I_2 _and BnMP-IIa**. Complete cDNA sequences were aligned using the Blastn program for multiple sequences alignments using as query BnMP-I_1 _sequence. Percentage similarity of different regions of the catalytic domain relatively to the query is shown. Gray boxes correspond to the spacer segment and the dashed boxes to the 3'UTR. Dots represent the gap corresponding to the RGD-disintegrin domain, represented in black in the P-II sequence. For the complete alignment, see additional file 2.

Additionally, our alignments have shown segments of approximately 12 bp, present in all sequences, complementary to or repeated in other regions of the same sequence or sequences from different classes (Figure 2, triangles and circles, respectively). Complementary segments were conserved in the different subclasses and were located enclosing the RGD motif or at the Zinc-binding site with its complement at the end of cysteine-rich domain. These complementary regions could be involved in eventual recombination within SVMPs of different types or even classes. This is further supported by evidences of recombination of P-I and P-II sequences shown in Figure 3. Alignments of cDNAs coding for catalytic domains of BnMP-I_1, _BnMP-I_2 _and BnMP-IIa showed that at the 5' catalytic region (0-246 bp), BnMP-I_1 _is identical to BnMP-IIa while at the same region BnMP-I_2 _presented 4% divergence. This region was followed by a segment of 121 bp where the three sequences were identical (246-365 bp). This region was denominated a "shift region" since after that we find an inversion of sequence identity. At the 3' catalytic region (365-630 bp), the complete identity was between BnMP-I_1 _and BnMP-I_2 _sequences while BnMP-IIa diverged 14% (Figure 3).

**Figure 4:**
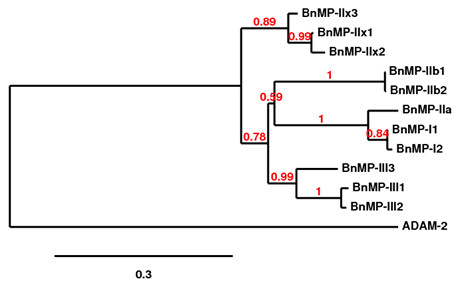
**Phylogetic representation of sequence alignments of cDNA regions coding for the mature proteins**. Trees were obtained as described in Methods section at http://www.phylogeny.fr. Numbers close to the nodes represent the support value for each branch.

Cladograms derived from cDNA alignments (region coding for mature protein) showed that class P-I (BnMP-I_1 _and BnMP-I_2_) and class P-III (BnMP-III_1_, BnMP-III_2 _and BnMP-III_3_) sequences were grouped in their own single branch. In opposition, sequences of class P-II (BnMP-IIa, BnMP-IIb_1,2 _and BnMP-IIx_1-3_) were distributed in distinct branches and BnMP-IIa segregated with BnMP-I group (Figure 4). Taken together, these observations show the heterogeneity of SVMPs class P-II and suggest that recombination between SVMPs may occur, explaining the identity observed between regions of SVMP sequences from classes P-I and P-II.

### Interclass recombination may be seen in other SVMPs

**Figure 5:**
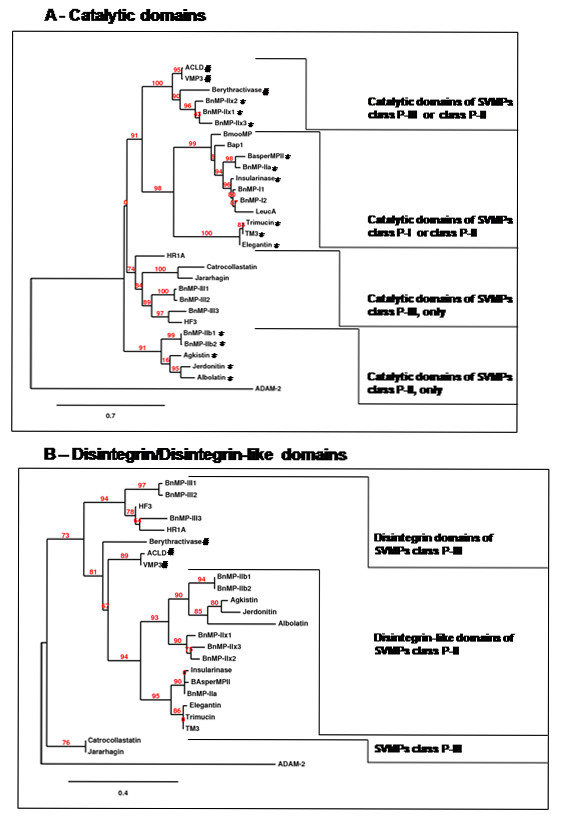
**Phylogetic representation of amino acid sequence alignments of independent domains**. Trees were obtained as described in Methods section at http://www.phylogeny.fr. Numbers close to the nodes represent the support value for each branch. Prior to phylogenetic analyses, cDNA sequences were translated and datasets were partitioned into a group with sequences of catalytic domains (amino acid residues at positions 1-214 of BnMP-III) or disintegrin/disintegrin-like domains (amino acid residues at positions 215-307 of BnMP-III). (*) indicates sequences of SVMPs class P-II; (#) indicates non-hemorrhagic class P-III SVMPs. The description, accession numbers and SVMP classification for other species sequences are shown in Table 2.

In order to evaluate if the transcripts described here would fit the nucleotide classification of SVMPs, BnMP protein sequences deduced from the cDNAs described above were aligned with sequences of SVMPs present in venoms of other viper snakes. As shown in Figure 5, independent alignments of catalytic (residues 1-214) or disintegrin/disintegrin-like domains (residues 215-307) resulted in distinct trees. In trees corresponding to catalytic domains (Figure 5A), four groups may be distinguished. One group included the catalytic domains of P-I and P-II SVMPs previously classified as P-Ia and P-IIa, respectively [11]; BnMP-I isoforms and BnMP-IIa were clustered into this group. Another cluster included only catalytic domains of P-II SVMPs which correspond to the non-processed forms classified as P-IIb, according to Fox and Serrano [11]; at this group we found BnMP-IIb sequences. The third group includes the catalytic domain of non-hemorragic class P-III SVMPs and, interestingly, BnMP-IIx isoforms (class P-II). Catalytic domains of P-III SVMPs linked to RGD disintegrin domain are not considered in the previous classification; therefore, we named these sequences as P-IIx. The last group included only SVMPs class P-III and BnMP-III isoforms (Figure 5A).

Phylogenetic inferences of disintegrin domains also showed a monophyletic distribution of disintegrin domain of P-II SVMPs (branch support value = 94%), further clustered in distinct branches. The first cluster included disintegrin domains from P-II sequences (P-IIa) and also BnMP-IIa that grouped with P-I SVMPs in the catalytic domain phylogeny; disintegrin domains of BnMP-II sequences type b were located together with other disintegrin domains of P-IIb SVMPs and disintegrin domains of P-IIx appeared as an independent branch sharing the same origin (branch support value = 93%) as P-IIb (Figure 5B). Distinct clusters included disintegrin-like domains of hemorrhagic or non-hemorrhagic P-III SVMPs. BnMP-III isoforms were included in the first cluster, together with hemorrhagic toxins, more specifically HR1a. Disintegrin domains from non-hemorragic were located closer to P-II disintegrin domains. However, in these cases, the statistical significance is low and a broader set of sequences would be necessary to evidence monophyly of P-III SVMP disintegrin domains.

**Figure 6:**
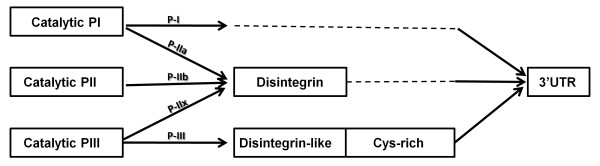
**Schematic representation of domain assembly possibilities between different classes of SVMPs**.

These observations suggest that there are at least three distinct precursors of class P-II SVMPs in venom gland and, in two of them, the catalytic domain may be shared with either P-I or P-III SVMPs. According to the high level of identity between sequences, these precursors might have been assembled by recombination between precursors of different classes of SVMPs. Evidences of high similarity between P-I and P-II cDNAs suggest post-transcriptional recombinations. Supporting this hypothesis, the 5' region of BnMP-I_1 _sequence (365 bp) was identical to BnMP-IIa isoforms (Figure 3). Considering that genes coding for SVMPs are subjected to accelerated evolution and that most of substitutions accumulate in coding regions, it is very unlike to expect 100% identity in cDNA regions of genes diverged previously, even though the time of divergence between P-I and P-II SVMPs is not reported in previous papers [8,24,25]. In this case, we hypothesize that the fragment of RNA coding for catalytic domain alternatively recombined with distinct 3's of SVMP transcripts, with or without the regions coding for the disintegrin domain, generating P-I or P-II SVMPs. The same hypothesis is not adequate to explain similarities among BnMP-IIx isoforms and class P-III catalytic domains. In this case, the 5' region was closely related but not identical to class P-III catalytic domains and alternative recombination with P-II disintegrin or P-III disintegrin-like/cysteine-rich domains are likely to have occurred at genomic level. The schematic representation of this hypothesis is shown in Figure 6.

The abundance of the different precursors of class P-II SVMPs in *B. neuwiedi *venom gland was then evaluated by qPCR. Specific primers for each type of BnMP-II sequence allowed quantifying **_~_**3.5 × 10^6 ^copies of BnMP-IIa, **_~_**5 × 10^5 ^copies of BnMP-IIb and **_~_**1.5 × 10^5 ^copies of BnMP-IIx per ng cDNA transcribed from the total venom gland RNA. According to these numbers, sequences coding for precursors that can be processed to generate class P-I SVMPs and free disintegrins are the most abundant in this group of P-II cDNAs.

### Distinct proteins are predicted by class P-II precursors

**Figure 7:**
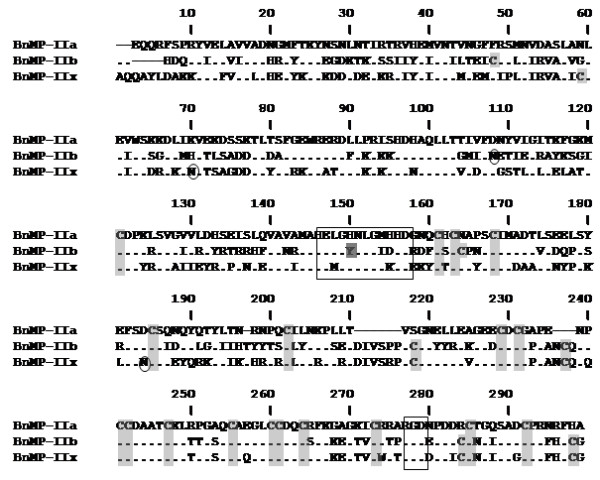
**Sequence alignment of class P-II SVMPs predicted by the cDNAs isolated from *B. neuwiedi *venom gland**. Sequences were aligned by ClustalW program. Cysteines are shadowed in gray; circles indicate the putative N-glycosylation sites; rectangles indicate the zinc-binding and RGD motif and dark gray the substitution by tyrosine of the histidine involved in zinc coordination.

Figure 7 shows the alignments of the putative proteins predicted by BnMP-II sequences. BnMP-IIa precursors code for sequences with the typical cysteine distribution of medium size disintegrins [26], with the RGD motif and no N-glycosylation sites. These precursors are very likely to be responsible for the generation of independent catalytic and disintegrin domains in the venom. BnMP-IIb and BnMP-IIx sequences also present disintegrin domains with RGD motifs but with additional cysteines at positions 218 (spacer region), 237, 284 and 298. Cysteines at positions 218/237 are also found in jerdonitin (GenBank: AAQ63966.1), agkistin (Swiss-Prot: Q8AWX7.1) and bilitoxin (Swiss-Prot: P0C6E3.1) P-II SVMPs and are frequent in P-III SVMPs [11]. These cysteines could be bound in order to provide stability for the proteins, avoiding the hydrolysis of the disintegrin domain. If this is true, it is reasonable to expect the presence of BnMP-IIb and BnMP-IIx in venoms in the unprocessed form (catalytic + disintegrin domains). However, cysteines at positions 292 and 306 were not found in any other disintegrins reported up to the moment. They may be involved in an extra S-S bound and give additional stability to the disintegrin domain. At catalytic domain, BnMP-IIx sequences differ from BnMP-IIb by two important aspects: they present an additional cysteine in the same position (59) found in berythractivase (GenBank: AAL47169.1) and two N-glycosylation sites at positions 70 and 191, which are found in several class P-III SVMPs. Catalytic domain of BnMP-IIb contain one additional pair of cysteines (positions 48 and 171) and a single N-glycosylation site at the same position (116) found in trigramin (GenBank: CAA35910.1) and flavoridin (GenBank: BAC00515.1) sequences (Figure 7). Interestingly, BnMP-IIb isoforms presented a substitution of histidines for tyrosines within the zinc-binding motif. These substitutions would impair the coordination of the zinc atom that is essential for catalysis, suggesting either that these cDNAs code for non functional enzymes or the proteins synthesized by these cDNAs may be used for further processing and generation of free disintegrins. This evidence was not due to experimental artifacts since the mutation responsible for H/Y substitution was detected in two independent RNA transcriptions and cloning.

## Discussion

Recently, transcriptomics and proteomics approaches have been extensively used to analyze the global composition of venoms from viper snakes. These studies provide a comprehensive knowledge of primary structure data based on sequences available in databanks. They are very important for an accurate description of the protein families expressed allowing evolutionary inferences about the generation of venom diversity and the toxin arsenal present in snake venoms [27,28]. In most of the referred studies, SVMPs appear as the major component in venoms of viper snakes and representatives of P-I, P-II and P-III classes were detected in all of them. However, these approaches are not the most appropriate for inferences of functional diversity since sequence characterization is usually based on molecular fragments of varying sizes that may omit structural rearrangements or motifs involved in important functions of a particular toxin. In this study, thirty-one cDNAs coding for mature SVMPs were completely sequenced from a gland of one specimen of *B. neuwiedi *snake. Internal alignments and phylogenetic inferences with databank SVMPs revealed new aspects of the evolution of functional diversity of this protein family not evidenced before by high throughput approaches.

Sequences of SVMPs from classes P-I, P-II and P-III were amplified from *B. neuwiedi *venom gland with particular abundance of cDNA sequences from class P-II SVMPs (BnMP-II). Representatives of each class were not identical, with at least two distinct copies of P-I cDNAs (with 98% identity) and three of P-III (with % identity values above 88). In opposition, great variability was observed amongst class P-II sequences with six distinct clones presenting a greater number of nucleotide substitution and divergence (identity from 73%). High sequence variability is not surprising since, considering that toxins evolved by accelerated evolution, a number of non-synonymous substitutions is predicted in coding regions driving to diversity of venom proteins, including SVMPs. Indeed, nucleotide substitutions were apparently required for the emergence of new functions as disclaimed for disintegrins [24]. Moreover, toxin genes contain in their exons a greater number of unstable triplets that were subjected to a faster point-mutation rate than constitutive genes [29]. In this regard, we suggest that point mutations are occurring in SVMPs with a higher frequency in representatives of class P-II, reflecting a higher plasticity in this class of genes that reflects in the generation of new functions in the venoms.

Besides the diversity of class P-II SVMPs generated by nucleotide substitutions, evidences of recombination between P-II disintegrin domain with catalytic domains of P-I or P-III SVMPs were also noted. When *B. neuwiedi *sequences were clustered by cladistic analysis, three distinct types of P-II sequences were detected with distinct branching: BnMP-IIa grouped with P-I SVMPs, BnMP-IIx with P-III, and BnMP-IIb in a third group, composed only of P-II SVMPs. This evidence suggests that recombination between genes encoding SVMPs might have occurred after the emergence of the primary gene copies coding for each scaffold. In their recent publication, Casewell et al. [25] suggest that different scaffolds of SVMPs were generated by minimization of genes with domain loss, which have occurred several times during evolution of this protein family. The loss of cysteine rich domain would have occurred once to generate P-II SVMPs and the loss of disintegrin domains would have occurred at least 8 times in P-II to generate P-I SVMPs. Indeed, this is a very plausible hypothesis already supported by other reports in the literature [8,24]. The data presented in this study is not in disagreement with this theory. However, we add an alternative mechanism to explain the diversity of precursors coding for SVMPs. The topology of the trees shown in this study indicates recombination of P-II genes with members from P-I and P-III classes both at DNA and RNA levels after the emergence of the three different scaffolds. The hypothetical recombination between P-I and P-II SVMPs might occur at the post-transcriptional level, supported by the striking identity (100%) between the 5'-terminal 365 bp fragment of BnMP-I and BnMP-IIa cDNA sequences that apparently were alternatively joined with two distinct fragments at 5', which diverged by nucleotide substitutions and the inclusion in only one of the fragments the region coding for the disintegrin domain. According to the previous papers [8,24,25], P-I SVMPs were derived from a PII ancestor and this would imply high similarity between such precursors. However, considering that genes coding for SVMPs are subjected to accelerated evolution and that most of substitutions accumulate in coding regions, it is very unlike to expect 100% identity in cDNA regions of genes that diverged previously, even though the time of divergence between P-I and P-II SVMPs is not reported in previous papers [8,24,25]. In this regard, post-transcriptional recombination is an appealing hypothesis to explain, together with genetic events, the generation of class P-I SVMPs in different events of SVMPs evolutionary history, as reported previously [25]. Corroborating this hypothesis we found complementary regions in *B. neuwiedi *cDNA sequences that could be involved in loop assembly in the RNAs enabling them for alternative trans-splicing leading to the formation of "hybrid" mRNAs. Evidences for alternative splicing have been shown in *Bungarus fasciatus *acetylcholinesterase gene generating different proteins in liver or venom gland [30]. This was also suggested by Siigur and colleagues [31], when studying the generation of diversity of serineproteinases from *Vipera lebetiba *venom and, more recently, Zeng et al. [32] provided evidences of trans-splicing for generation of toxin diversity in the venom of *Buthus martensii *scorpion. Alternative splicing may explain the intriguing distribution of class P-I SVMPs in previous phylogenetic studies of SVMPs, also grouped with class P-IIa SVMPs in different branches of their phylogenetic tree [25].

The other evidence of recombination between different classes of SVMPs was deduced from the phylogenetic inferences of independent domains. Several studies report the phylogenetic relationships of SVMPs isolated from closely-related species, or of specific classes or domains [8,23,24,33-36]. Usually, these studies deal with a large number of sequences with different degrees of similarity. In this study, the hypothesis of interclass recombination arose from experimental data, during sequence alignments. Thus, the analyses were carried out using a different approach driven to identify such recombination in other snake species. For this purpose, we selected by megablast [37] only the most similar sequences (>85% identity) of independent domains from each BnMP sequences and carried out the phylogenetic analysis with this selected group of toxins. This approach may not represent the evolutionary history of the protein family, but allowed the characterization of closest relationships between catalytic domains from different classes of SVMPs, with statistically supported clusters enclosing P-I and P-IIa (99%) or including P-IIx and P-III (100%) catalytic domains. Considering the disintegrin domain, P-IIa, P-IIb and P-IIx sequences were monophyletic (94%). We understand that this is strong evidence that the multiple domain structure observed in SVMPs has undergone different evolutionary history, and the protein variability of this protein family may be dependent on recombination at different points of the evolution of this protein family. In the case of BnMP-IIx and P-III SVMPs, recombination events might occur at the genomic level with the assembly of P-II disintegrin domains with catalytic regions from genes of non-hemorrhagic SVMPs class P-III. The possibility that genomic recombination of independent domains occurs by exon shuffling cannot be ruled out since this is an accepted mechanism for evolution of modular molecules, particularly plasma and extracellular matrix proteins [38,39]. In venoms, segment switches in exons (ASSETS) have been demonstrated and correlated to targeting diversification [40]. Moreover, it has also been reported by different authors the occurrence of SVMP structures that might have been assembled by P-III catalytic domain with P-IIa disintegrin domain [41] or by PIIa catalytic domains with P-III disintegrin-like domains lacking the cysteine-rich domain [42]. These evidences supporting recombination between P-III catalytic and P-II disintegrin domains indicate that the loss of cysteine rich domain may be a more frequent mechanism and may have occurred in different events during evolution of SVMPs. Unfortunately, these mechanisms of recombination are still speculative since, up to the moment, genomic sequences coding for SVMPs were not completely disclosed and the exon/intron distribution at catalytic domain is still unknown.

Regardless of the mechanisms used for generation of new genes/mRNAs encoding class P-II SVMPs, this is certainly the class under highest adaptative pressure and positive selection. Soto et al. [36] showed that accumulation of mutations is less frequent in class P-III SVMPs than in class P-II. In this study, we noted in a single snake three independent P-II structures, coding for distinct proteins. BnMP-IIa sequence shares catalytic domain with P-I SVMPs and representatives enclosed in this cluster generate, by processing, the P-I SVMPs (only catalytic domains) and the classical RGD-disintegrins; BnMP-IIb sequences grouped with other class P-IIb SVMPs corresponding to the non-proteolytic processed P-II SVMPs; BnMP-IIx group corresponds to a new subclass of P-II SVMP precursor which predicts proteins with catalytic domain of non-hemorrhagic P-III SVMPs and a disintegrin domain with a higher number of cysteines, an extra pair at C-termini that may be involved in a new disulfide bond reported here for the first time. This domain association has not been reported yet and the presence of these molecules in venoms in the unprocessed form is still unknown and the possibility that this precursor is a pseudogene is not ruled out up to the moment. This distribution is in agreement to the gene tree reported by Casewell et al. [25] with a few differences: the authors include a fourth group with dimeric P-IId SVMP and did not evidence the P-IIx SVMPs. It is important to note the large number of sequences enclosed on their P-I/P-IIa group. In our case, BnMP-IIa was the most abundant cDNA in the gland of *B. neuwiedi*, but in the pool of *B. neuwiedi *venom, the most abundant proteins are two isoforms of a class P-I SVMP, BnP1 and BnP2, which were partially sequenced revealing approximately 90% identity with either BnMP-I or BnMP-IIa cDNAs [43]. Taken together, the great number of P-I/P-IIa SVMPs described and their high expression levels in venom glands point to the advantage of this form of precursor since it generates both P-I SVMPs and free disintegrins from a single precursor. The possible recombination between P-I and P-II precursors shown in this study is of great relevance for the evolution of SVMPs and the generation of the functionally active representatives of this family of proteins.

## Conclusions

A great diversity was observed in cDNAs encoding SVMPs in a single venom gland of a *B. neuwiedi *specimen. The most complex group was the class P-II represented by three independent subclasses with evidences that they may be arisen by recent recombination either at genomic level or post-transcription between different classes of SVMPs. This includes a new subclass of P-II SVMP precursor, P-IIx, composed of a combination of P-III catalytic domain and a RGD disintegrin domain with extra cysteines at C-termini. These evidences add new possibilities of SVMPs scaffolds by the inclusion of a P-IIx type of SVMP. In regard to biosynthesis of the P-I group, at least in our set of precursors, P-I proteins appeared to be synthesized from both P-I and P-II precursors, and it is very likely that P-I precursors were derived from P-II post-transcriptionally. Moreover, our data enlarge the possibilities of mechanisms involved in generation of diversity of this class of toxins. However, a complete understanding of the genesis of venom diversity and definite evidences of recombination are still dependent on the resolution of the genome of a viper snake.

## Methods

### Amplification and sequencing of SVMP cDNAs

A specimen of *Bothrops neuwiedi *snake was collected at the city of Itu, São Paulo, Brazil (23°15'56″S and 47°17'56″W). After quarantine, the snake was milked to stimulate mRNA synthesis and 4 days later euthanized with CO_2 _for extraction of the venom glands. Gland tissue was homogenized with Trizol (Invitrogen) and total RNA isolated using the RNAEasy Lipid Tissue mini kit (Qiagen). Double stranded cDNA was generated by reverse transcription with Superscript III (Invitrogen) and oligo(dT) as primers. SVMP cDNAs were amplified by PCR using Platinum^® ^Taq DNA Polymerase High Fidelity (Invitrogen) with specific primers able to amplify P-I, P-II and P-III classes. The forward primer (5'CCAAAATGTGTGG(A/G)GTAACT 3') corresponded to bases coding for the cysteine-switch motif present in P-I, P-II and P-III pro-domains which is approximately 150 bp upstream the N-terminal of mature SVMPs (PRODOM) and the reverse primer (5'CCAAAATGTGTGG(A/G)GTAACT 3') was designed from a conserved region at the 3'UTR sequence, 80 bp downstream the stop codon, also common to P-I, P-II and P-III classes (3'UTR). PCR amplification consisted of 30 cycles of 94°C (30 s), 55°C (1 min), and 68°C (1 min). A final extension step was performed for 7 min, at 68°C. PCR cDNA products were separated by 1.5% agarose gel electrophoresis, and purified using the Gelclean Extraction Kit (Promega). Amplification products were independently cloned into pGEM-T Easy Vector (Promega) and transfected into *E. coli *DH5α. Ten colonies were randomly selected from each transformation tube, their plasmids were amplified and submitted to cDNA sequencing using the ABI Prism Big Dye Terminator Cycle Sequence Ready Reaction kit and the ABI 3100 sequenator (Applied Biosystems). Inserts from selected clones were completely sequenced in both directions (plus and minus strands) with at least three independent readings from each cDNA region.

### Quantification of copy numbers of BnMP-II cDNAs

The copy numbers of BnMP-II cDNAs were determined by absolute quantitative real-time PCR [44] in the Line Gene K Thermal Cycler (Hangzhou Bioer Technology Co.) using the *fqdpcr-4.2.20 *software. Briefly, cDNA copy number reverse transcribed from isolated mRNA was estimated based on calibration curves of absolute number of plasmids containing either BnMP-IIa, BnMP-IIb or BnMP-IIx cDNAs. The real-time qPCR was performed using a mixture of 12.5 μL Master Mix - Sybr Green Rox Plus (LGC Biotechnology), 200 ng cDNA, 170 nM of each primer (P-IIa FW (5' GAACAACAAAGATTCT 3'), P-IIa RV (5' AAATCTGCACTGGTC 3'), P-IIb FW (5' CATGACCAAAGATACATTGA 3'), P-IIx FW (5' GCACAACAAGCATACTTG 3') and P-IIbx RV (5' GGACCAAA(T/C)TTCTA 3') and RNAse free water adjusted to 25μL final volume. The following thermal cycling protocol was used for all primers: 15 min at 95 °C followed by 40 cycles of 15 s at 95 °C, 30 s at 60 °C and 30 s at 72 °C. After the amplification, the melting curve analysis was performed with a temperature gradient from 65 to 90 °C at 1 °C/min and the resulting band visualized in 1.5% agarose gels. The cDNA copy numbers were the average of triplicates of each sample.

### SVMP sequences from databanks

Nucleotide and amino acid SVMP sequences were retrieved from the National Center for Biotechnology Information database http://www.ncbi.nlm.nih.gov. Due to the great availability of SVMP sequences in databanks and the frequency of partial length sequences, in order to detect the closest relationships between SVMPs we selected for our study only complete sequences and the most similar to each domain characterized in *B. neuwiedi *SVMPs. For that, we carried out independent searches for highly similar nucleotide sequences using the Megablast Algorithm and the non-redundant nucleotide collection [37] taking as query sequence catalytic, disintegrin, disintegrin-like or cysteine-rich domains coding regions of each BnMP cDNA sequence. Sequences with domain identity higher than 80% and query coverage higher than 90% were selected excluding partial length cDNAs. The sequences selected by each independent domain were then reunited in a single dataset, used for the phylogenetic inferences as nucleotide sequences or after translation using the Translate Tool algorithm at ExPASy http://expasy.ch/tools/dna.html. The description, accession numbers and SVMP classification of each sequence is shown in Table 2.

**Table 2:** SVMP protein sequences used for phylogenetic inferences

Protein	Description	SVMP Class	Accession
**BmooMP**	***Bothrops moojeni ***zinc metalloproteinase BmooMPalfa-I	**P-I**	P85314

**LeucA**	***Bothrops leucurus ***zinc metalloproteinase leucurolysin-A	**P-I**	P84907

**BaP1**	***Bothrops asper ***hemorrhagic metalloproteinase BaP1	**P-I**	P83512.2

**Insularinase**	***Bothrops insularis ***insularinase precursor mRNA	**P-II**	AY736107.1

**BasperMPII**	***Bothrops asper ***type II metalloproteinase mRNA	**P-II**	DQ247725.1

**Agkistin**	***Gloydius halys zinc ***metalloproteinase-disintegrin agkistin	**P-II**	AAL60587.1

**Jerdonitin**	***Trimeresurus jerdonii ***jerdonitin mRNA	**P-II**	AY364231.1

**Albolatin**	***Trimeresurus albolabris ***metalloproteinase homolog disintegrin albolatin	**P-II**	P0C6B6.1

**Trimucin**	***Protobothrops mucrosquamatus***pro-trimucin	**P-II**	CAA54364.1

**TM3**	***Protobothrops mucrosquamatus ***trimutase precursor	**P-II**	AAB94016.1

**Elegantin**	***Trimeresurus elegans ***elegantin-1a precursor mRNA	**P-II**	AB059571.1

**HF3**	***Bothrops jararaca ***hemorrhagic metalloproteinase HF3 mRNA	**P-III**	AF149788.5

**HR1a**	***Trimeresurus flavoviridis ***hemorrhagic metalloproteinase HR1a	**P-III**	Q8JIR2.1

**Catrocollastatin**	***Crotalus atrox ***catrocollastatin precursor	**P-III**	AAC59672.1

**Jararhagin**	***Bothrops jararaca ***zinc metalloproteinase-disintegrin jararhagin	**P-III**	CAA48323.1

**ACLD**	***Agkistrodon contortrix laticinctus ***zinc metalloproteinase-disintegrin ACLD	**P-III**	AAC18911.1

**VMP3**	***Agkistrodon contortrix laticinctus ***metalloproteinase VMP-III precursor mRNA	**P-III**	GQ451435.1

**Berythractivase**	***Bothrops erythromelas ***berythractivase (ery1) mRNA	**P-III**	AF450503.1

**ADAM-2**	**Human**	**root**	GI55743079

### Sequence Alignments and Phylogenetic Inferences

Protein or cDNA sequences were aligned using ClustalW [45]. Alternatively, Blastn program for multiple sequences alignments [37] was used for alignments of *B. neuwiedi *nucleotide sequences. Phylogenetic trees were set based on either nucleotide sequence or the amino acid sequence alignments, which combined protein-derived sequences and DNA-translated amino acid sequences. Phylogenetic analyses were run at *http://www.Phylogeny.fr*as described [46]. Sequences were aligned using the MUSCLE program [47] according to the *κ*mer distances clustered by UGPMA. The Gblocks program [48] was used to eliminate poorly aligned positions and divergent regions. PhyML 3.0 was used for phylogenies [49] including substitution models HKY85 for DNA and WAG for proteins. The ALTR test (SH-like) was used to access the support values of each branch [50]. In some cases, prior to phylogenetic analyses, datasets were partitioned into three groups: 1-complete SMVP (catalytic, disintegrin/disintegrin-like and cysteine-rich domains); 2-catalytic domains (amino acid residues at positions 1-214 of BnMP-III); 3-disintegrin/disintegrin-like domains (amino acid residues at positions 215-307 of BnMP-III).

## Authors' contributions

Conceived and designed the experiments: GSM, RHV, AMMS. Performed the experiments: KFG, MSF, MCC, PBC, JAPJ. Analyzed the data: GSM, PBC, RHV, AMMS. Wrote the paper: GSM, PBC, JAPJ, RHV, AMMS. All authors read and approved the final manuscript.

## Supplementary Material

Additional file 1 **Sequence alignment of cDNAs encoding *B. neuwiedi *SVMPs**. Complete cDNA sequences were aligned using the Blastn program for multiple sequences alignments using as query BnMP-III_1 _sequence: (.) identical nucleotides to the first sequence; (-) gaps introduced to maximize sequence alignments; regions coding for functional motifs as the zinc-bindin residues and RGD disintegrin tripeptide are boxed; alignments of complementary (-) or repeated (+) regions in relation to BnMP-III_1 _sequence are highlighted in gray. Click here for file

Additional file 2 **Sequence alignment of cDNAs encoding BnMP-I_1_, BnMP-I_2 _and BnMP-IIa**. Complete cDNA sequences were aligned using the Blastn program for multiple sequences alignments using as query BnMP-I_1 _sequence: (.) identical nucleotides to the first sequence. Note the absence of disintegrin domain coding region in BnMP-I_1 _and BnMP-I_2 _sequences. Click here for file
