# Supplementary material for: Diversity of metalloproteinases in Bothrops neuwiedi snake venom transcripts: evidences for recombination between different classes of SVMPs
Source: BMC Genet. 2011 Nov 1;12:94. doi: 10.1186/1471-2156-12-94 (PMC3217872; doi:10.1186/1471-2156-12-94)
Supplement: Additional file 1 — Sequence alignment of cDNAs encoding B. neuwiedi SVMPs. Complete cDNA sequences were aligned using the Blastn program for multiple sequences alignments using as query BnMP-III1 sequence: (.) identical nucleotides to the first sequence; (-) gaps introduced to maximize sequence alignments; regions coding for functional motifs as the zinc-bindin residues and RGD disintegrin tripeptide are boxed; alignments of complementary (-) or repeated (+) regions in relation to BnMP-III1 sequence are highlighted in gray. [file 1471-2156-12-94-S1.PDF]

```
BnMP-III1    4     CAGCAAAGATATAACCGCTACAAATACGTTGAGCTTCTTATAGTTGCAGACTACAAAATG  63
BnMP-III2    1     ..T.............A......T............A.......................  60
BnMP-III3    4     ................C................G..G..................G....  63
BnMP-IIb1    12                           ....A.......CG.C...........TC...G....  48
BnMP-IIb2    12                           ....A.......CG.C...........TC...G....  48
BnMP-IIx1    28                         ............T..G.CG.....CTG...C.TGG....  66
BnMP-IIx2    28                         ............T..G.CG.....CTG...C.TG.....  66
BnMP-IIx3    28                         ............T..G.CG.....CTG...C.TGG....  66
BnMP-IIa     24                           .............GC.G..........TA..GG....  60
BnMP-I1      24                           .............GC.G..........TA..GG....  60
BnMP-I2      22                         ......A........GC.G..........TC..GG....  60

BnMP-III1    64    GTCACGAGAAACAATGGCAATTTAGATGAGATAAGAACAAAAATATATGAAATTGTCAAC  123
BnMP-III2    61    ............................................................  120
BnMP-III3    64    .......A....................................................  123
BnMP-IIb1    49    TA...A.A.T..G.A..TG..AA.AC.A.......TT...T...................  108
BnMP-IIb2    49    TA...A.A.T..G.A..TG..AA.AC.A.......TT...T...................  108
BnMP-IIx1    67    T....AGT.T....A.A.G........A......A.........................  126
BnMP-IIx2    67    TA..AA.A.T....A.A.G...............A..G..G...................  126
BnMP-IIx3    67    T....AGT.T....A.A.G........A......A.........................  126
BnMP-IIa     61    T......A.T....CA........A..ACT.........CGGG..C.......G......  120
BnMP-I1      61    T......A.T....CA........A..ACT.........CGGG..C.......G......  120
BnMP-I2      61    T......A.T....CA.....G..A..ACT.........TGGG..C.......G......  120

BnMP-III1    124   AATGTAAATGTGATTTTCAGATATTTGTACATTCGTATAGCACTGGTTGCCATAGAAATT  183
BnMP-III2    121   ................G...........................................  180
BnMP-III3    124   TT.T....C.A......................................G..........  183
BnMP-IIb1    109   .T.T...C..A.....G.....C....A.T......G....T.......G.C........  168
BnMP-IIb2    109   .T.T...C..A.....G.....C....A.T......G....T.......G.C........  168
BnMP-IIx1    127   .C.A.G....A...G....TCCCG...A.T......G.....T..A..TG.C........  186
BnMP-IIx2    127   .C.A.G....A...G....TCCC....A.T......G.....T..A..TG.C........  186
BnMP-IIx3    127   .C.A.G....A...G....TCCCG...A.T......G.....T..A..TG.C........  186
BnMP-IIa     121   .C........G.T.........C.A..A.TG..GA.GC.T......C.AA.C.....G..  180
BnMP-I1      121   .C........G.T.........C.A..A.TG..GA.GC.T......C.AA.C.....G..  180
BnMP-I2      121   .G.C......G.T.........C.A..A.TG..GA.GC.T........AA.C.....G..  180

BnMP-III1    184   TGGTCCAACGGAGATTTGATTAATGTGGAGTCAGCAGCAAATGTTACTTTGGACTCATTT  243
BnMP-III2    181   ............................................................  240
BnMP-III3    184   .........A.................ACA......T..GG..A................  243
BnMP-IIb1    169   .......G.......C....GC.C...ACA.T.T.....G...A.........TG.....  228
BnMP-IIb2    169   .......G.......C....GC.C...ACA.T.T.....G...A.........TG.....  228
BnMP-IIx1    187   ......G..A.....AA......C...ACA.......G.G...........AG.......  246
BnMP-IIx2    187   ......G..A.....AA......C...ACA.......G.G...A......AT........  246
BnMP-IIx3    187   ......G..A.....AA......C...ACA.......G.G...........AG.......  246
BnMP-IIa     181   ........GAA.........C..A......AA..ATT...G.AAA......ACG......  240
BnMP-I1      181   ........GAA.........C..A......AA..ATT...G.AAA......ACG......  240
BnMP-I2      181   ........GAA.........C..A......AA..ATT...G.AAA......ACG......  240

BnMP-III1    244   GGAACCTGGAGAGAGAAAGACTTGCTGAAACGCAAAAGTCACGATAACGCTCAGTTACTC  303
BnMP-III2    241   ............................................................  300
BnMP-III3    244   .............G..C...T.......GG..G........T.....T............  303
BnMP-IIb1    229   ...GAA..........G...T..T.....G.......AA..T...C.T............  288
BnMP-IIb2    229   ...GAA..........G...T..T.....G.......AA..T...C.T............  288
BnMP-IIx1    247   A...AA.......C..C...T........G...........T.....T............  306
BnMP-IIx2    247   A...AA.......C..C...T........G...........T.....T............  306
BnMP-IIx3    247   A...AA.......C..C...T........G...........T.....T............  306
BnMP-IIa     241   ...GAA..........G...T......CCT....T......T...C.T............  300
BnMP-I1      241   ...GAA..........G...T......CCT....T......T...C..............  300
BnMP-I2      241   ...GAA..........G...T......CCT....T......T...C..............  300
```

```
BnMP-III1   304   ACGGCCATTGACTTCAATGGACCAAC--AATAGGAATAGCTTAC----GTGGCCAGCATG   357
BnMP-III2   301   .....................T..--...............----...........    354
BnMP-III3   304   ..............AG.........--T..........A.A----A.A...G.A...   357
BnMP-IIb1   289   ....G...GAT........A.A...T--TGA.....GG......AAAA...----.T..A  342
BnMP-IIb2   289   ....G...GAT........A.A...T--TGA.....GG......AAAA...----.T..A  342
BnMP-IIx1   307   ....TG........TG.....T....--TT.....T.GA..CG.----A.A....C....  360
BnMP-IIx2   307   ....TG........TG.....T....--TT.....T.GA..G.A----C......C....  360
BnMP-IIx3   307   ....TG........TG.....T....--TT.....T.GA..CG.----A.A....C....  360
BnMP-IIa    301   ...A......T....G..--.ATT..GTT.........A..A.G----T.C.G..AA...  354
BnMP-I1     301   ...A......T....G..CA..A...--T........G......----ACA...G.....  354
BnMP-I2     301   ...A......T....G..CA..A...--T...............----ACA...G.....  354


BnMP-III1   358   TGCGACCCGAAGCGTTCTGTAGGAGTTGTCATGGATTATAG--CT-TATCAAATTTTGTG  414
BnMP-III2   355   ........A...................CA........--..-......G.......   411
BnMP-III3   358   ...A.A.T...CGAA..............CA.........--.C-C.AT..G.......  414
BnMP-IIb1   343   .....................A....TCG.........AA..AG.CGTC......--   400
BnMP-IIb2   343   .....................A....TCG.........AA..AG.CGTC......--   400
BnMP-IIx1   361   ..T.....AT.TG.........C.A.AA.TGA.T..CG...--.C-C.AT....C...A.  417
BnMP-IIx2   361   ..T......T.T..........C.A.AA.TGA.T..CG...--.C-C.AT....C...A.  417
BnMP-IIx3   361   ..T......T.T..........C.A.AA.TGA....C....--.A-..AT..G.C..AA.  417
BnMP-IIa    355   ..T.........T..............TT.....C....--TG-A.AT..G.C..CA.  411
BnMP-I1     355   ..........GC.AA.......C......T......C....--TA-A.AA....A..CG.  411
BnMP-I2     355   ..........GC.AA.......C......T......C....--TA-A.AA....A..CG.  411


                                    Zn-binding
BnMP-III1   415   CTTGCAGTTATAATGGCCCATGAGATGGGTCATAATCTGGGCATGGATCATGACACAGGT  474
BnMP-III2   412   ............................................................  471
BnMP-III3   415   G......................................AAT..................  474
BnMP-IIb1   401   -.....AA..G..........C.....T.............T..........G..A.   459
BnMP-IIb2   401   -.....AA..G..........C.....T.............T...T......G..A.   459
BnMP-IIx1   418   G....T.................................A.A.....TGA.AAG    477
BnMP-IIx2   418   G....T.................................A.A.....TGA.AAG    477
BnMP-IIx3   418   A.G....................................A.A.....TGA.AAG    477
BnMP-IIa    412   G........GC..........C.................C.......TGG.AA.    471
BnMP-I1     412   G.........C..........C.............................---.A.    468
BnMP-I2     412   G.........C..........C.............................---.A.    468


BnMP-III1   475   TCCTGTTCTTGTGGTGGTTACTCATGTATTATGTCATCTGGGA---TGAGCCATCAACCT  531
BnMP-III2   472   ...............................TC......---..............   528
BnMP-III3   475   .T...............C................TC...A..---.A..TG...C....  531
BnMP-IIb1   460   .T.........CT..CC.A.......C......---G...AC.CAG.A..TG........  516
BnMP-IIb2   460   .T.........CT..CC.A.......C......---G...AC.CAG.A..TG........  516
BnMP-IIx1   478   .A....A.....AA..C.........C......GATG.C.CAC---.A...A..T.T...  534
BnMP-IIx2   478   .A....A.....AA..C.........C......GATG.C.CAC---.A...A..T.T...  534
BnMP-IIx3   478   .A....A.....C.A..C.ACG.....C......G.TG.C.CAG---.A..A...G.T.G.  534
BnMP-IIa    472   CAG...CA...CAA..C.CC.......C......---G...AC.CAC.A...G.AG...T.  528
BnMP-I1     469   A.....A....C....C.A.G...........G.T..CACA.---.A...A.AGG..T.  525
BnMP-I2     469   A.....A....C....C.A.G...........G.T..CACA.---.A...A.AGG..T.  525


BnMP-III1   532   TCCAAACTATTCAGCGATTGTAGTTA----TATCCAATATTGGCTCTTTATTA-TGAAGC  586
BnMP-III2   529   ..........................----..............G..........-.....  583
BnMP-III3   532   .....C..........A........A.----GCAGGC....CA.AGG.A.....-ATGTTT  586
BnMP-IIb1   517   ....GT.GG...............AT----.GAT.......T.GGA.A.....T.C.TA.  572
BnMP-IIb2   517   ....GT.GG...............AT----.GAT.......T.GGA.A.....T.C.TA.  572
BnMP-IIx1   535   ........G......A........C.GGAG....A..----..AAG.A.C...-.A..A.  589
BnMP-IIx2   535   ........G......A........C.GGAG....A..----..AAG.A.C...-.A..A.  589
BnMP-IIx3   535   ........G......A........A.----G.AAG...G.CA.ACG.A.C...-.A..A.  589
BnMP-IIa    529   ...T.CGAG...............C.----G.AT......CA.ACG.A.C...-CT..T.  583
BnMP-I1     526   ...TTTGAG...............C.----G.AT......CA.ACG.A.G...-CT....  580
BnMP-I2     526   ...TTTGAG...............C.----G.AT......CA.ACG.A.G...-CT....  580
```

```
BnMP-III1    587   AGAAACCACAA--TGCATTCTCAATGAACCCTTGAGAACAGATATTGTTTCACCTCCAGT   644
BnMP-III2    584   ...........--...............................................   641
BnMP-III3    587   .T.........--...............................................   644
BnMP-IIb1    573   TT.TTAT....GT...C..TA....A....A.C.GAG.......................   632
BnMP-IIb2    573   TT.TTAT....GT...C..TA....A....A.C.GAG.......................   632
BnMP-IIx1    590   .T.G.....G.--...C........AGG.........................G......   647
BnMP-IIx2    590   .T.G.....G.--...C........AGG.........................G......   647
BnMP-IIx3    590   .T.G.......--..............G...............G.........G......   647
BnMP-IIa     584   GT..C.....G--............A........CT..------------------....   623
BnMP-I1      581   .T..C......--............A........CT..------------------....   620
BnMP-I2      581   .T..C......--............A........CT..------------------....   620


BnMP-III1    645   TTGTGGAAATGAACTTTTGGAGATGGGAGAAGAATGTGACTGTGGCTCTCCTAGAAATTG   704
BnMP-III2    642   ............................................................   701
BnMP-III3    645   .....................................................C......   704
BnMP-IIb1    633   ......C...T.CTA.CG.....A.........T............C.....GC......   692
BnMP-IIb2    633   ......C...T.CTA.CG.....A.........T............C.....GC......   692
BnMP-IIx1    648   .....................G.......................C.....GC......  707
BnMP-IIx2    648   ......................G.......................C.....GC......   707
BnMP-IIx3    648   ......................G.......................C.....GC......   707
BnMP-IIa     624   ..C...................GC......................G.....-.-----   677
BnMP-I1      621   ..C...................GC.......T..............G.....          672
BnMP-I2      621   ..C...................GC.......T..............G.....          672


BnMP-III1    705   TCGAAATCCATGCTGCGATGCTGCTACGTGTAAACT-ACACCCATGGGTAGAGTGTGAAT   763
BnMP-III2    702   ......................A.............-.......................   760
BnMP-III3    705   ....T....G.....T........A..C........-...GT..................   763
BnMP-IIb1    693   ..AG...........T........A...........G...-A..G.ATC.C......C.G   751
BnMP-IIb2    693   ..AG...........T........A...........G...-A..G.ATC.C......C.G   751
BnMP-IIx1    708   ..A......G.............TA..C........G...-...G..TC.C......C.C   766
BnMP-IIx2    708   ..A.....AG..............A...........G...-...G..TC.C......C.C   766
BnMP-IIx3    708   ..A.....AG..............A..A........G...-...G..TC.C......C.G   766
BnMP-IIa     678   ---......G..............A..C........-GAGA...G...CGC......C.G   733


BnMP-III1    764   CTGGAGAGTGTTGTGGCCAGTGCAGATTTATAAAAACAGGAAATGTATGCAGGCCACAA   822
BnMP-III2    761   ...................................G.......................   819
BnMP-III2    857                ............C..                                842(-)
BnMP-III3    764   ..............................A.GG.C...C.TCA.A....CA.G..GC.   822
BnMP-III3    860                ............C..                                845(-)
BnMP-IIb1    752   AA...CT........A...........C...AG...GA.....CA......C..A...C.   810
BnMP-IIb2    752   AA...CT........A...........C...AG...GA.....CA......C..A...C.   810
BnMP-IIx1    767   AA...CT.C......A...A...........AG...GA.....CA......T..AG..C.   825
BnMP-IIx2    767   AA...CT........A...A...........AG...GA.....CC......T..AG.AC.   825
BnMP-IIx3    767   A.....T........A...............G...GA.....CA......T..AG..C.   825
BnMP-IIa     734   AA...CT........A...............AGGG.G.......AA.....C..AG.GC.   792
BnMP-IIa     827                ...........                                    816(-)


                   Disintegrin
BnMP-III1    823   AGGAGTGAGTGTGACATTGCTGAAAGCTGCACTGGCCAATCTGCTCAGTGTCCCACAGATG   883
BnMP-III2    820   .............................................................   880
BnMP-III2    789                          ...T............                      774(-)
BnMP-III3    823   ...............................................G.........T.....   883
BnMP-III3    792                          ...T............                      777(-)
BnMP-IIb1    811   ...G....---...GCCG.A...TT......A....AT.......G.C.......G.TT.C   868
BnMP-IIb1    786                  ..T..T..............T..                       765(-)
BnMP-IIb2    811   ...G....---...GCCG.A...TT......A....AT.......G.C.......G.TT.C   868
BnMP-IIb2    786                  ..T..T..............T..                       765(-)
BnMP-IIx1    826   ...G....---....CCG.A.A.TT......A....AT.......GGC.......G.TT.C   883
BnMP-IIx2    826   ...G....---....CCG.A.ATTT......A....AT.......GGC.......G.TT.C   883
BnMP-IIx3    826   ...G....---....CCG.A.A.TT......A....AT.......GGC.......G.TT.C   883
BnMP-IIa     793   ...G....---.A..CCG.A...TC...................G.C.......G.A..C   850
```

```
BnMP-III1    884   ACTTCCATAAGAATGGACAACCATGCCTATCTAACTACGGTTACTGTTACAATGGGAATT   943
BnMP-III2    881   ..............................................C.............   940
BnMP-III3    884   .....A.A.G...................CA...............C.............   943
BnMP-IIb1    869   .....                                                          873
BnMP-IIb2    869   .....                                                          873
BnMP-IIx1    884   .....                                                          888
BnMP-IIx2    884   .....                                                          888
BnMP-IIx3    884   .....                                                          888
BnMP-IIa     851   G.......                                                       858

BnMP-III1    944   GCCCCATCATGCATCACCAATGTTATGCTCTCTTTGGTTCAGGTGCAACTGTGGCTCAAG   1003
BnMP-III2    941   ................................................T...........   1000
BnMP-III3    944   ...........T..............A...........G...A..T..........G...   1003

BnMP-III1    1004  ATGGATGTTTTAAATTTAATGACAGAGGGGATAAATTTTTCTACTGCAGAAAGGAAAATG   1063
BnMP-III2    1001  ............................................................   1060
BnMP-III3    1004  .............TGC......A.A.......G...A.......................   1063

BnMP-III1    1064  TTATAATTACTCCGTGTGCACAAGAGGATGTAAAGTGTGGCAGGTTATTCTGTCATACTA   1123
BnMP-III2    1061  ............................................................   1120
BnMP-III3    1064  G.G...A..T...A......A................................G...A..   1123
BnMP-IIx1    759                ...........                                       769(+)
BnMP-IIx2    759                ...........                                       769(+)

BnMP-III1    1124  AGAAGTCTGAATGCGATTTTGATTATTCAGTAGACCCAGATTATGGAATGGTTGATCATG   1183
BnMP-III2    1121  ............................................................   1180
BnMP-III2    452                                     ...........                  442(-)
BnMP-III3    1124  ......A.CC....C...ACA.....................T..............T..   1183
BnMP-III3    455                                     ...........                  445(-)
BnMP-IIx1    458                                     ...........                  448(-)
BnMP-IIx2    458                                     ...........                  448(-)
BnMP-IIx3    458                                     ...........                  448(-)
BnMP-IIa     452                                     ...........                  442(-)
BnMP-I1      452                                     ...........                  442(-)
BnMP-I2      452                                     ...........                  442(-)

BnMP-III1    1184  GAACAAAATGTGCAGATGGAAAGGTCTGCAACAGCAACAGGCAGTGTGTTGATGTGACTA   1243
BnMP-III2    1181  ............A...G...........................................   1240
BnMP-III3    1184  ............................................................   1243
BnMP-IIb1    890             ..G........T........---...........................   936
BnMP-IIb2    890             ..G........T........---...........................   936
BnMP-IIx1    905             ..G........T........---...........................   951
BnMP-IIx2    905             ..G........T........---...........................   951
BnMP-IIx3    905             ..G........T........---...........................   951
BnMP-IIx3    760          ............                                            771(-)
BnMP-IIa     872             ..G........T........---...........................   918
BnMP-I1      681                                  ...................             699
BnMP-I2      681                                  ...................A.........   709

BnMP-III1    1244  CAGCCTACTAATCAACCACTGGCTTCTCTCAGATTTGAT   1282
BnMP-III2    1241  .......................................   1279
BnMP-III3    1244  .......................................   1282
BnMP-IIb1    937   .................T.....................   975
BnMP-IIb2    937   .................T.....................   975
BnMP-IIx1    952   ..........G......T.....................   990
BnMP-IIx2    952   .................T.....................   990
BnMP-IIx3    952   ..........G......T.....................   990
BnMP-IIa     919   .........----..........................   953
BnMP-I2      710   .......A.........T.....................   748
```
