# Supplementary material for: Diversity of metalloproteinases in Bothrops neuwiedi snake venom transcripts: evidences for recombination between different classes of SVMPs
Source: BMC Genet. 2011 Nov 1;12:94. doi: 10.1186/1471-2156-12-94 (PMC3217872; doi:10.1186/1471-2156-12-94)
Supplement: Additional file 2 — Sequence alignment of cDNAs encoding BnMP-I1, BnMP-I2 and BnMP-IIa. Complete cDNA sequences were aligned using the Blastn program for multiple sequences alignments using as query BnMP-I1 sequence: (.) identical nucleotides to the first sequence. Note the absence of disintegrin domain coding region in BnMP-I1 and BnMP-I2 sequences. [file 1471-2156-12-94-S2.PDF]

```
BnMP-I1     1     AAAATGTGTGGGGTAACTGAGACTAATTGGGAATCATATGAGCCCATCAAAAAGGCCTCT  60
BnMP-I2     1     ....................................G...........G...........  60
BnMP-IIa    1     ...........A................................................  60

BnMP-I1     61    CAGTCAAATCTTACTCCTGAACAACAAAGATTCTCCCCAAGATACGTTGAGCTTGCTGTA  120
BnMP-I2     61    ............................A...........A....A..............  120
BnMP-IIa    61    ............................................................  120

BnMP-I1     121   GTTGCAGATAACGGAATGTTCACGAAATACAACAGCAATTTAAATACTATAAGAACACGG  180
BnMP-I2     121   .........C.............................G.................T..  180
BnMP-IIa    121   ............................................................  180

BnMP-I1     181   GTACATGAAATGGTCAACACTGTAAATGGGTTTTTCAGATCTATGAATGTTGATGCATCA  240
BnMP-I2     181   ...................G.C......................................  240
BnMP-IIa    181   ............................................................  240

BnMP-I1     241   CTGGCTAACCTAGAAGTTTGGTCCAAGAAAGATTTGATCAAAGTGGAGAAAGATTCAAGT  300
BnMP-I2     241   ....T.......................................................  300
BnMP-IIa    241   ............................................................  300

BnMP-I1     301   AAAACTTTGACGTCATTTGGAGAATGGAGAGAGAGAGATTTGCTGCCTCGCATAAGTCAT  360
BnMP-I2     301   ............................................................  360
BnMP-IIa    301   ............................................................  360

BnMP-I1     361   GATCACGCTCAGTTACTCACGACCATTGTCTTCGATCAACAAACTATAGGAATGGCTTAC  420
BnMP-I2     361   .....................................................A......  420
BnMP-IIa    361   .....T..............................A.TT.CGT.........AA..A.G  420

BnMP-I1     421   ACAGCCGGCATGTGCGACCCGAGCCAATCTGTAGCAGTTGTTATGGATCATAGTaaaaaa  480
BnMP-I2     421   ............................................................  480
BnMP-IIa    421   TTC.G.AAA.....T.......AG.TT.......G.......T...........G...T.  480

BnMP-I1     481   aaTATTCGGGTTGCAGTTACAATGGCCCATGAGCTGGGTCATAATCTGGGCATGGATCAT  540
BnMP-I2     481   ............................................................  540
BnMP-IIa    481   .G.C...A..........G...................................C.....  540

BnMP-I1     541   GACGATACCTGTACTTGCGGTGCTAAGTCATGTATTATGGCTTCCACAATAAGCAAAGGA  600
BnMP-I2     541   ............................................................  600
BnMP-IIa    541   ..T...CAG...CA....AA....CCC.....C.........GA....C.....G...A.  603
                           \
                           |
                          GAA

BnMP-I1     601   CTTTCCTTTGAGTTCAGCGATTGTAGTCAGAATCAATATCAGACGTATGTTACTAAGCAT  660
BnMP-I2     601   ............................................................  660
BnMP-IIa    604   .......AC.......................................C.......T.G.  663

BnMP-I1     661   AACCCACAATGCATTCTCAATAAACCCTTGCTAACAGTTTCTGGAAATGAACTTTTGGAG  720
BnMP-I2     661   ............................................................  720
BnMP-IIa    664   ........G...................................................  723

BnMP-I1     721   GCGGGAGAATAATGTGACTGTGGCGCTCCTGAA                             740
BnMP-I2     721   .................................                             740
BnMP-IIa    724   .........G.......................AATCCGTGCTGCGATGCTGCAACCTGT  770

BnMP-I1
BnMP-I2
BnMP-IIa    771   AAACTGAGACCAGGGGCGCAGTGTGCAGAAGGACTGTGTTGTGACCAGTGCAGATTTAAG  830

BnMP-I1
BnMP-I2
BnMP-IIa    831   GGAGCAGGAAAAATATGCCGGAGAGCAAGGGGTGATAACCCGGATGATCGCTGCACTGGC  890

BnMP-I1     741                                                               .  741
BnMP-I2     741                                                               .  741
BnMP-IIa    891   CAATCTGCTGACTGTCCCAGAAATCGCTTCCATGCCTAACCAACAATGGAGATGGAATGG  950

BnMP-I1     752   .......................                                       764
BnMP-I2     752   .......................A................A.AATC....T.........  801
BnMP-IIa    951   TCTGCAGCAACAGGCAGTGTGTTGATGTGACTACAGCCTACT----AACCACTGGCTTCT  996

BnMP-I1     802   ............   813
BnMP-I2
BnMP-IIa    997   CTCAGATTTGAT   1018
```
